# The prevalence and determinants of blood pressure control among hypertension patients in eastern Zimbabwe: A cross-sectional study

**DOI:** 10.1371/journal.pone.0293812

**Published:** 2024-03-07

**Authors:** Paddington Tinashe Mundagowa, Pemberai Zambezi, Priscillah Muchemwa-Munasirei

**Affiliations:** 1 Africa University Clinical Research Center, Africa University, Mutare, Zimbabwe; 2 Department of Epidemiology and Biostatistics, Arnold School of Public Health, University of South Carolina, Columbia, SC, United States of America; 3 College of Social Sciences, Theology, Humanities and Education, Africa University, Mutare, Zimbabwe; University of Zimbabwe Faculty of Medicine and Health Sciences, ZIMBABWE

## Abstract

**Background:**

Uncontrolled blood pressure (BP), also known as hypertension, is a leading cause of morbidity and mortality globally. Lowering the elevated BP can significantly reduce one’s risk for cardiovascular diseases. This study aimed to ascertain the determinants of BP control among hypertension patients.

**Methods:**

The data analyzed were from the exploratory survey of the Home Management of Hypertension (HoMHyper) project in eastern Zimbabwe. Hypertension patients were selected from the Chronic Disease Registers of five public health clinics using simple random sampling. A pretested interviewer-administered questionnaire was used to collect data, and the patient’s BP was measured. The primary outcome, BP control, was used as a categorical variable (controlled vs. uncontrolled) to conduct a bivariate analysis. Variables significant at p<0.2 were included in the multivariable logistic regression analysis to control for confounding. Statistical significance in the final model was set at p<0.05.

**Results:**

Data from 321 hypertension patients were analyzed; their mean age was 62.3±11.9 years. The prevalence of controlled BP was 41.4% (95% Confidence interval-CI = 36.0%-46.9%). After adjusting for confounding, patients’ residence and medication stocks were associated with BP control. Patients who resided in high-density suburbs had higher odds of uncontrolled BP than those who resided in middle- and low-density suburbs (Adjusted odds ratios-AOR = 2.5; 95% CI = 1.4–4.4; p<0.01). Hypertension patients who experienced medication stockouts over the last six months had higher odds of uncontrolled BP than patients who did not experience stockouts (AOR = 1.8; 95% CI = 1.1–2.9).

**Conclusion:**

BP control among hypertension patients was suboptimal. Patient residence and antihypertensive medication stockouts were independent predictors of blood pressure control. We recommend exploring sustainable financing through private-public partnerships to ensure the availability of subsidized antihypertensive medication.

## Introduction

Globally, an estimated 1.3 billion people aged between 30 to 79 years are living with elevated blood pressure (BP), also known as hypertension [1]. Hypertension is defined as the persistently high force of blood flowing through the blood vessels with systolic blood pressure (SBP) greater than 140mmHg and diastolic blood pressure (DBP) greater than 90mmHg [2]. Uncontrolled hypertension is associated with coronary heart disease, heart failure, and renal disease [3, 4]. Despite the robust evidence reporting the primacy of maintaining low BP on significant cardiovascular disease risk reduction [5, 6], the global burden linked to hypertension is on the increase [7].

Typically, BP is checked for both out-patients and in-patients, and the primary goal is early hypertension detection, assessing the effectiveness of treatment, estimating cardiovascular risk, and informing clinical decision-making [8]. In 2017, a large-scale BP Measurement Month Campaign in which more than 1.2 million BP measurements were conducted among adults revealed that approximately 17.3% with elevated BP were not treated, and 46.3% had uncontrolled BP [9]. Because patients with elevated BP are often asymptomatic, clinicians must be vigilant in checking BP for all adult patients despite their hypertension status. A 5mmHg reduction in DBP can lower the risk of stroke by approximately 34% and ischemic heart disease by 21% [10], while a 10mmHg reduction in SBP can lower the incidence of stroke by 41% [11]. Patients on antihypertensive medication should check their BP regularly to improve treatment outcomes. Routine monthly clinic visits may be insufficient since BP varies substantially over time [12]. In addition, recent BP management guidelines recommended home-based BP management [6, 13–15], and this chronic disease management model can improve and sustain BP control in low-resource contexts such as the sub-Saharan African (SSA) region [16].

The determinants of hypertension control have been widely investigated and understanding these factors among middle-aged and older adults is vital considering that hypertension is more prevalent, and BP control is difficult to achieve for patients of these age groups [17]. Old age, alcohol use, black ethnicity, obesity, and multiple comorbidities were reported to have an association with a lack of BP control [18, 19]. Suboptimal hypertension control is also linked to lower socioeconomic status [20] and this may be attributed to limited access to care, differential treatment, increased chronic stress, and lower health literacy among the poor. Other possible pathways linked to suboptimal BP control include pathophysiology, disease management, and individual risk factor profile [18].

In 2016, the World Health Organization (WHO) launched a package to enhance BP control and the package was made up of five main domains namely, diagnosis (measurement accuracy and CVD risk evaluation), treatment (standardized protocols and intensification), follow-up and continuity of care, delivery system and system performance evaluation [21]. Despite having this guide, disparities in hypertension control persist with high-income countries (HICS) having four times the hypertension control rate compared to low-and middle-income countries (LMICs) [22]. The observed improved control in HICs was mainly attributed to the role of antihypertensive treatment [23]. Thus, LMICS can adopt and prioritize successful models from HICs to improve BP control among patients.

Without active screening and focused intervention implementation, the SSA region’s hypertension burden is projected to worsen from 74.7 million in 2013 to 125.5 million in 2025 [24] and 216.8 million by 2030 [25]. The average hypertension prevalence in SSA is 31% [26], only 11% of hypertension patients are controlled, and adherence to prescribed antihypertensives is estimated at 60% [27]. This is mainly due to a dearth of knowledge on evidence-based guidelines, a lack of commitment from policymakers [27] and poor access to diagnostic and antihypertensive treatment services in low- and middle-income countries (LMICs) such as Zimbabwe is poor [28].

The government of Zimbabwe acknowledged the growing importance of noncommunicable diseases (NCDs) and set them among the priorities in health service provision in the National Health Strategy (2020–2025) [29]. Although this is a step toward action, the Ministry of Health and Child Care has no standardized Community Health Worker (CHW)-delivered program for hypertension and the proposed work will be a pilot program in an urban setting. The public health sector has the most extensive infrastructure to support healthcare systems. However, the National Health Budget is poorly funded, resulting in limited expenditure on NCDs. In 2021, the allocation for health spending was low (13%) against the 15% recommended at the Abuja Declaration target, and only 3% of the Public Health Program budget was dedicated towards NCDs [30].

Research studies conducted in Mutare, Zimbabwe revealed poor adherence to recommended lifestyle medication and hypertension treatment, high rates of uncontrolled BP, and poor hypertension patient management [31–33]. These studies were all conducted at a local tertiary referral hospital, and evidence specific to hypertension patients seen at the primary health level is lacking. Local data is vital to target resources as well as plan and evaluate interventions. As part of the exploratory work to implement a community-based intervention for hypertension management, we conducted a survey to ascertain the prevalence and determinants of BP control among hypertension patients in Mutare Urban.

## Methods

### Study design and site

The data used in this study were from the exploratory survey of the Home Management of Hypertension (HoMHyper) project, an implementation science study on Community Health Worker (CHW)-delivered BP measurements and lifestyle modification education among hypertension patients in Mutare, Zimbabwe. Mutare is in Manicaland Province on the eastern border between Zimbabwe and Mozambique, and it is the country’s third most populous city covering an area of about 191.2km^2^. In 2022, the city had an estimated population of 224,802 [34]. Three public hospitals mainly attended referrals from Manicaland Province, while eight public primary care health clinics and an estimated 22 private medical facilities focused on outpatient health services for most of the city residents. The study was conducted at the five primary public health clinics only and these were Chikanga, City, Dangamvura, Florida, and Hobhouse Polyclinics. Manicaland Province has high poverty prevalence levels estimated at 71% [35]. Chikanga, Dangamvura, and Hobhouse residential areas are primarily high-density suburbs while Florida and the catchment areas around the City clinic are mostly low- and middle-density suburbs.

### Study population and sampling

The study population was hypertension patients (recorded from May 1, 2022, to April 30, 2023) selected from the Chronic Diseases Registers at the clinics using the simple random sampling method. After retrieving the line list from the register, all patients were allocated a number and the RAND function in Microsoft Excel was used to select a predetermined sample for each clinic. The patient’s contact or that of their next of kin was retrieved from the register, and local CHWs were briefed about the survey, provided with a recruitment script, and delegated by the facility to call the selected patients. Across the clinics, the patient response rate ranged from 84% (City Clinic) to 95% (Chikanga Clinic). The CHWs physically visited selected patients whose phone contacts were unreachable using the registered address. We included hypertension patients aged 30 to 80 years who had received hypertension-related services (monthly routine check-ups and medication refills) from the local clinic, were diagnosed at least six months before the survey, were on antihypertensive treatment, and had records confirming their medical history. We restricted participant recruitment to 30 to 80 years because global trends show a relatively low hypertension prevalence before the age of 30 years and threshold and treatment target guidelines differ in older ages [36]. The demographic was selected because it comprises the most affected patients and will likely benefit from the planned intervention. Pregnant women, mentally, and terminally ill patients were excluded from the study. Pregnancy increases the risk of hypertension and the threshold and management of hypertension in pregnancy varies widely from nonpregnant individuals [37].

The Cochran’s formula [n = Z^2^(p(1-p))/e^2^] where n = sample size, Z = test statistic at 95% confidence interval, p = proportion of sample with the attribute of interest, and e = desired precision (0.05) was used to determine the sample size of study participants, assuming that the proportion (p) of uncontrolled hypertension among hypertension patients was 24.6% [38]. The adequate sample size was 318 hypertension patients after adjusting for 10% nonresponse.

#### Data collection

The study was conducted using survey questions developed after an extensive literature review on factors influencing BP control in a resource-limited setting. The survey questions included demographic, comorbidity, current hypertension management, treatment adherence, and health system measures. Interviews were conducted by three research assistants with at least a bachelor’s degree and a research nurse. The nurse and research assistants were trained for data collection by the principal investigator (PTM) and a Research Administrator from Africa University. The interviewer-administered survey was pretested on 18 hypertensive patients at a non-participating public clinic. Two-masked linguistic experts from Africa University translated survey questions into the Shona language. Study participants were interviewed at the local clinic.

The selected participants were informed to visit the clinic for BP measurement within three days before the survey appointment. Two BP measurements were done at most ten minutes apart and the nurse recorded the average. Another two BP measurements were done on the day of data collection, and the averages of the two BP measurements conducted on the two separate days were used to ascertain BP Control. To control for white coat hypertension, the study goal and BP measurement procedure were explained to the patient and questions were answered before the procedure was conducted. The patients were allowed to rest for at least five minutes and the research assistants took this time to give a health talk. Patient BP was measured by the research nurse using calibrated Omron 10 Series Upper Arm BP Monitors and employing the recommended steps for correctly measuring BP [8, 39]. Data were collected from June 4 to July 7, 2023. Data collection was approved by the Africa University Research Ethics Committee (Approval number: AU2668/22) and the Medical Research Council of Zimbabwe (Approval Number: MRCZ/A/2977), and permission to conduct the study was sought from the Mutare City Health Director’s Office. All participants provided written informed consent that was signed in duplicate, and the participant kept one copy and the interviewer kept the other copy for filing purposes. Thump prints were used for participants who could not read and write and the nurse-in-charge at the clinic signed the informed consent form as a witness.

### Measures

#### BP control

Blood pressure control was based on the Joint National Committee (JNC) 8 Guidelines [40], and hypertension grading was done using the European Society of Cardiology and the European Society of Hypertension Guidelines [15]. The guidelines recommend a BP control target and threshold of an SBP <140mmHg and a DBP <90mmHg for patients under 60 years and an SBP <150 mmHg and a DBP <90mmHg for patients 60 years and older. Hypertension was classified into normal (SBP <140 and DBP <90mmHg), Grade 1 (SBP = 140–159 or DBP = 90-99mmHg), Grade 2 (SBP = 160–179 or DBP = 100-109mmHg), and Grade 3 (SBP≥180 or DBP≥110mmHg).

#### Demographics and current hypertension management

Analyses included seven demographic variables: age, sex, level of education, religion, estimated monthly personal income, medical insurance, residence, and the clinic. Age was based on completed years, while level of education was based on the Zimbabwe National Census classifications. Religion was derived from the nature of the doctrine followed, and medical insurance was categorical (yes/no). Participant residence was classified according to stand sizes as per reported street and residential suburb. These ranged from high density (70–200 m^2^), middle density (300–500 m^2^), and low density (800–2000 m^2^) as stipulated in Circular Number 70, 2004 of the Ministry of Local Government, Public Works and National Housing [41]. Patients were asked about comorbidities (the interview also checked the patient’s medical records) and variables on current BP management, including owning a BP machine (yes/no), affording to buy a BP machine (yes/no), ability to interpret a BP reading (yes/no), and medication stockouts were defined as missing the prescribed medication for at least two consecutive days (yes/no). Health system-related variables were frequency of clinic visits, requiring assistance to visit the clinic (yes/no), the primary source of hypertension-related services (public clinic/hospital/pharmacy or private clinic/hospital/pharmacy), antihypertensive medication refills received over the last six months, date BP was last checked, and distance from the clinic.

### Data analysis

Summary descriptive statistics were reported as frequencies, percentages, means, and standard deviations. The primary outcome, BP control, was used as a categorical variable (controlled vs. uncontrolled) to conduct a bivariate analysis. All variables significant at p<0.2 during the bivariate analysis were included in the multivariable logistic regression analysis to control for confounding. Statistical significance in the final model was set at p<0.05 and the Hosmer-Lemeshow test was used to assess the model’s goodness of fit. Participants with missing outcome variables were excluded from the analysis. Data analysis was conducted in SAS® version 9.4.

## Results

Three hundred thirty-three hypertension patients were interviewed, and the response rate was 94.3%. Twelve participants were excluded from the analysis because they had missing data on the primary outcome variable (BP readings). The mean age of the hypertension patients was 62.3±11.9 years. Participants were female (74.1%), identified as Protestant Christians (75.7%), earned less than USD$50 a month (56.7%), had no medical insurance (67.9%), were from high-density suburbs (66.0%), and had comorbidities (57.0%). The overall mean distance between the place of residence and the clinic was 2.1±1.8 km (Table 1). The mean monthly personal income was USD$65.0±84.5, and the most common comorbidities were diabetes mellitus (24.8%) and HIV (24.8%). The pooled mean years since hypertension diagnosis was 13.5±10.6 years, and there was no significant difference between the mean year after diagnosis among BP-controlled (13.8±10.7) and uncontrolled patients (13.2±10.6), p = 0.85.

**Table 1:** Characteristics of study participants (n = 321).

Variable	Characteristic	Frequency (n)	Percentage (%)
Clinic	Florida	61	19.0
	Chikanga	74	23.0
	Hobhouse	78	24.3
	Dangamvura	60	18.7
	City	48	15.0
Residence	High-density suburbs	212	66.0
	Middle-/low-density suburbs	109	34.0
Age (years)	30–39	11	3.4
	40–49	45	14.0
	50–59	61	19.0
	60–69	103	32.1
	70–79	79	24.6
	80	22	6.9
Sex	Male	83	25.9
	Female	238	74.1
Highest level of formal education	None	18	5.6
	Primary	120	37.4
	Secondary	152	47.3
	Tertiary	31	9.7
Religion	Pentecostal	51	15.9
	Protestant	243	75.7
	Apostolic	22	6.8
	African tradition	5	1.6
Monthly income (USD$)	0–49	182	56.7
	50–99	55	17.1
	100–199	48	15.0
	200–299	26	8.1
	≥300	10	3.1
Had medical insurance	Yes	103	32.1
	No	218	67.9
Distance between residence and clinic (km)	≤ 1.0	153	47.7
	1.1–2.0	79	24.6
	2.1–3.0	37	11.5
	>3.0	52	16.2
Comorbidities	Hypertension only	138	43.0
	Had comorbidities	183	57.0
Had a BP machine	Yes	57	17.8
	No	264	82.2
Affords to buy a BP machine	Yes	68	21.2
	No	253	78.8
Needs assistance to visit clinic	No	270	84.1
	Yes	51	15.9
Frequency of clinic visits	Weekly	13	4.0
	fortnightly	23	7.2
	Monthly	208	64.8
	Quarterly	77	24.0
Experienced medication stockouts 6 months before survey	Yes	101	31.5
	No	220	68.5
Solution to stockouts^a^	Borrow from others	35	34.6
	Missed dose	64	63.4
	Used herbs	2	2.0
Can interpret BP reading	Yes	170	53.0
	No	151	47.0
The primary source of BP services	Public clinic/hospital	276	86.0
	Private clinic/hospital/pharmacy	45	14.0
BP last checked	Last 2 weeks	97	30.2
	This month	61	19.0
	Last month	97	30.2
	≥2months ago	66	20.6
Medication refills received from the clinic in the last 6 months	None	166	51.7
	1 month	103	32.1
	2 months	9	2.8
	≥3 months	43	13.4

^a^n = 101; BP = blood pressure

### Current BP management

Approximately 17.8% of the participants reported that they had no personal BP machines, and when asked if they could afford to buy a BP machine, 78.8% responded “No”. 84.1% did not need assistance to visit the clinic, and most patients (64.8%) visited the clinic monthly. More than a third of patients (34.6%) had experienced antihypertensive medication stockouts over the last six months, and of these, 63.4% had missed doses, while 34.6% borrowed from others. About 53% were able to interpret a BP reading, 86% used public health services as the primary source of BP management services, and 51.7% did not receive any antihypertensive medication refills from the clinic over the past six months (Table 1). The mean age of hypertension patients with controlled BP (64.2±11.3 years) was significantly higher than that of patients with uncontrolled BP (60.8±12.1 years), p = 0.04 after controlling for the number of years since diagnosis. There were no significant differences in monthly income among controlled (USD$56.9±92.1) and uncontrolled patients (USD$70.7±78.5), p = 0.15. The mean SBP and DPB were 149.7±22.3mmHg and 81.3±12.1mmHg. The prevalence of controlled BP was 41.4% (95% Confidence interval-CI = 36.0% - 46.9%). The pooled mean estimated distance from the clinic to the patient’s residence was 2.1±1.8 km.

### Factors associated with BP control

Tables 2 and 3 display the bivariate and multivariable logistic regression analyses of the factors associated with BP control among hypertension patients in Mutare. The crude analysis revealed that age (Odds ratio-OR = 1.6; 95% CI = 1.0–2.5; p = 0.04), residence (OR = 0.3; 95% CI = 0.2–0.5; p<0.01), level of education (OR = 0.5; 95% CI = 0.3–0.9; p<0.01), and need for assistance to visit the clinic (OR = 1.5; 95% CI = 0.8–2.7; p = 0.01) were statistically significant at p<0.05 level.

**Table 2:** Bivariate analysis of the factors associated with BP control among hypertension patients in Mutare urban (n = 321).

Variables	Characteristics	Uncontrolled n (%)	Controlled n (%)	COR (95% CI)	p-value
Age (years)	30–64	105 (64)	59 (36)	1.6 (1.0–2.5)	0.04*
	≥65	83 (53)	74 (47)	1.0	
Residence	High density	106 (50)	106 (50)	0.3 (0.2–0.5)	<0.01*
	Middle- and low-density	82 (75)	27 (25)	1.0	
Highest level of education	None/primary	69 (50)	68 (50)	0.5 (0.3–0.9)	<0.01*
	Secondary or higher	119 (65)	64 (35)	1.0	
Sex	Male	49 (59)	34 (41)	1.0 (0.6–1.7)	0.92
	Female	139 (58)	99 (42)	1.0	
Religion	Apostolic/African religion	17 (63)	10 (37)	1.1 (0.5–2.5)	0.83
	Other	171 (58)	123 (42)	1.0	
Monthly income ($USD)	<200	163 (57)	122 (43)	0.6 (0.3–1.2)	0.16
	≥200	35 (69)	11 (31)	1.0	
Had medical insurance	Yes	67 (65)	36 (35)	1.5 (0.9–2.4)	0.11
	No	121 (56)	97 (44)	1.0	
Distance between residence and clinic (km)	>2	61 (69)	28 (31)	1.3 (0.7–2.1)	0.39
		147 (63)	85 (37)		
Comorbidities	Yes	82 (59)	56 (41)	1.1 (0.7–1.7)	0.79
	No	106 (58)	77 (42)	1.0	
Had BP machine	Yes	36 (63)	21 (37)	1.3 (0.7–2.3)	0.44
	No	152 (58)	112 (42)	1.0	
Affords to buy a BP machine	Yes	46 (68)	22 (32)	1.6 (0.9–2.9)	0.09
	No	142 (56)	111 (44)	1.0	
Can interpret BP reading	Yes	103 (61)	67 (39)	1.2 (0.8–1.9)	0.44
	No	85 (56)	66 (44)	1.0	
Needs assistance to visit clinic	No	166 (61)	104 (39)	2.1 (1.1–3.9)	0.01*
	Yes	22 (43)	29 (57)	1.0	
Experienced medication stock outs 6 months before survey	Yes	47 (47)	54 (54)	0.5 (0.3–0.8)	<0.01*
	No	141 (64)	79 (36)	1.0	
Primary source of BP services	Public clinic/hospital	161 (58)	115 (42)	0.9 (0.5–1.8)	0.83
	Private clinic/hospital	27 (60)	18 (40)	1.0	
Medication refills received from the clinic in the last 6 months	None	99 (60)	67 (40)	1.1 (0.7–1.7)	0.69
	At least 1 month’s supply	89 (57)	66 (43)	1.0	

BP: blood pressure; CI: Confidence interval; COR: Crude odds ratios

**Table 3:** Crude and adjusted odd ratios for the factors associated with BP control among hypertension patients in Mutare urban.

Variable	COR (95% CI)	p-value	AOR (95% CI)	p-value
Residence (high density vs. middle- and low- density)	0.3 (0.2–0.5)	<0.001*	2.5 (1.4–4.4)	<0.01*
Age (30–64 vs. ≥64) years	1.6 (1.0–2.5)	0.04*	0.8 (0.5–1.4)	0.48
Highest level of education (none/primary vs. secondary/higher)	0.5 (0.3–0.9)	<0.01*	1.6 (0.9–2.7)	0.1
Experienced medication stockouts six months before survey (yes vs. no)	0.5 (0.3–0.8)	<0.01*	1.8 (1.1–2.9)	0.03*
Needs assistance to visit clinic (no vs. yes)	2.1 (1.1–3.9)	0.01*	0.7 (0.4–1.4)	0.30
Had medical insurance (yes vs. no)	1.5 (0.9–2.4)	0.11	0.9 (0.5–1.5)	0.69
Afford to buy a BP machine (yes vs. no)	1.6 (0.9–2.9)	0.09	0.8 (0.4–1.5)	0.50
Monthly income (<$200 vs. ≥$200)	0.6 (0.3–1.2)	0.16	0.7 (0.3–1.6)	0.38

Hosmer-Lemeshow Chi-square value = 3.44, p = 0.90; AOR: Adjusted odds ratios; BP: blood pressure; CI: Confidence Interval; COR: Crude odds ratios

After controlling for variables that were significant in the bivariate analysis, patients’ residence was found to be associated with BP control (Adjusted odds ratios-AOR = 2.5; 95% CI = 1.4–4.4; p<0.01). Patients residing in high-density suburbs were 2.5 times more likely to have uncontrolled BP compared to patients who resided in middle- and high-density suburbs. Hypertension patients who experienced antihypertensive medication stockouts last six months before the survey were 1.8 times more likely to have uncontrolled BP compared to patients who did not experience antihypertensive medication stockouts (AOR = 1.8; 95% CI = 1.1–2.9).

## Discussion

Uncontrolled BP is a significant risk factor for cardiovascular-related morbidity and mortality, and BP management is a major challenge in LMICs, including Zimbabwe. This study aimed to determine the factors influencing BP control among hypertension patients in Mutare Urban. The prevalence of BP control among the interviewed patients was 41.3%, while the mean SBP and DPB were 149.7±22.3mmHg and 81.3±12.1mmHg, respectively. Residing in high-density suburbs and experiencing medication stockouts six months before the survey were associated with higher odds of uncontrolled BP.

The result showing 41.3% prevalence of controlled BP among hypertensive patients was consistent with other similar studies conducted in LMICs such as Ethiopia (42%) [42, 43], South Africa (41.9%) [44], Iran (38.3%) [45], and India (46.9%) [46], and Zimbabwe (41.4%) [31]. However, this was low when compared to other similar studies in Kenya (48.6%) [47] and Zimbabwe (52%) [32]. The differences in the prevalence may be due to one of the studies being population-based [47], and the findings were from analyzing a large dataset while the other was based on one facility [32]. Considering the consequences of hypertension, this control rate should ideally be 100%. The low BP control rates could be linked to the reported high incidences of medication stockouts. It is concerning to note that during the six months before the survey, more than half of patients did not receive a refill from the clinics, while 86% acknowledged that their primary source of hypertension-related services was public clinics, hospitals, or pharmacies. There is a need to improve the essential services given to hypertension patients, in particular, medication refills to improve BP control among patients.

This study revealed increased odds of uncontrolled BP among patients living in high-density suburbs when compared to middle-/low-density suburbs. Rapid urbanization in Zimbabwe resulted in overcrowding and informal settlements associated with poor living conditions, poor access to healthcare services, low-income groups, and extreme poverty [48]. Suboptimal BP control is prevalent among the lowest-income groups [49]. Given the complexity of the associations between income disparities and BP control, targeting the provision of access to treatment only may be inadequate to overcome inequality [20]. Preventive measures must account for the socioeconomic paradigms when designing BP management interventions.

Experiencing antihypertensive medication stockouts six months before the survey was associated with uncontrolled BP. According to in-depth interviews conducted by the authors of this study among the clinic nurses and local health authorities in the same setting (manuscript submitted to another journal), the only consistently available medication at the public clinic was Hydrochlorothiazide. The majority of hypertension patients in Zimbabwe are on two or more antihypertensive medications, with the most prescribed medications being Angiotensin Converting Enzyme Inhibitors or Angiotensin Receptor Blockers, [50] thus, could not access these medications from the public clinics. Hypertension patients in low-income settings have limited access to hypertension treatment and if available, the prices are beyond the reach of the majority [51].

Faced with a limited choice of antihypertensive medications at public clinics, patients on other medication regimens are compelled to seek services from private for-profit pharmacies. The private pharmacies in the country inflate prices since they are not bound by the Essential Drug List of Zimbabwe guidelines [50]. The possibility of ending up buying cheaper counterfeit medication from unregistered pharmacies is high. WHO estimates that 50% of essential drugs in SSA are counterfeit [52]. Another possible explanation for the low rate of BP control is poor adherence to lifestyle modification behaviors and medication in Mutare [31]. More intensive efforts are required to enhance BP control among hypertension patients. We recommend advocating for additional public funding to fill the gap in the medication supply chain to improve the availability of antihypertensive medications in the public health sector. Our findings provide an impetus to evaluate patient access to affordable hypertension treatment in the public sector in Zimbabwe. Private partners and non-profit organizations could play a significant role in boosting the medication supply chain.

### Study strengths and limitations

Using a random sampling method to select the study participants increased patient representation and may have helped reduce selection bias. An average of two BP readings measured at most three days apart, using a calibrated sphygmomanometer, one nurse, while observing the WHO guidelines for BP measurement, improved rigor and reduced measurement bias to ascertain BP control among patients. However, the survey was based on self-report, which can be influenced by reporting bias. Data were collected from public health clinics only. Although these clinics were the primary sources of health services for patients who could not afford private health services; we could have obtained richer data by including patients from private health facilities. The study did not include some key confounding variables, including anthropometric and behavioral measurements. In addition, environmental and climatic factors that may affect the control of BP were not assessed. Causal inferences were not possible since we employed the cross-sectional study design.

## Conclusion

BP control among hypertension patients was relatively low. Patients’ residence and medication stockouts influenced BP control with living in high-density suburbs and patients who had experienced antihypertensive medication stockouts had higher odds of having uncontrolled BP. Intervening through a sustainable supply of antihypertensive medication may improve BP control among patients in this setting. This will be pivotal to avert the costly cardiovascular complications that can lead to disability and premature mortality. Most patients depend on the public health sector, and we recommend accounting for the socioeconomic status of hypertension patients when intervening. Long-term availability of medication is contingent on sustainable funding mechanisms through private-public partnerships involving the government, private sector, donors, and nongovernmental organizations to fill the medication supply gap. Lastly, evidence-based implementation approaches could be used to assist BP control and management among patients in this and other similar settings.

## Supporting information

S1 Appendix (XLSX)
